# Association of estimated glucose disposal rate with erectile dysfunction: mediating role of neutrophil-to-lymphocyte ratio

**DOI:** 10.29219/fnr.v70.14300

**Published:** 2026-08-28

**Authors:** Hesong Pan, Feng Chen, Lulu Pan

**Affiliations:** The Wenzhou Third Clinical Institute Affiliated to Wenzhou Medical University, Wenzhou People’s Hospital, Wenzhou city, China

**Keywords:** estimated glucose disposal rate, obesity, erectile dysfunction, insulin resistance, diabetes mellitus

## Abstract

**Background:**

Insulin resistance (IR) has been established as an independent risk factor for erectile dysfunction (ED).

**Objective:**

This study aimed to investigate the association between the estimated glucose disposal rate (eGDR) – a simple and non-invasive surrogate measure of IR – and the prevalence of ED.

**Design:**

This cross-sectional study analyzed data from the National Health and Nutrition Examination Survey (NHANES) conducted between 2001 and 2004. The association between ED prevalence and eGDR was assessed using receiver operating characteristic analysis, multivariate logistic regression, restricted cubic spline (RCS) regression, and subgroup analysis. In addition, the mediating role of the neutrophil-to-lymphocyte ratio (NLR) in the eGDR–ED relationship was evaluated through mediation analysis.

**Results:**

A total of 3,774 subjects were included, of whom 1,072 (28.4%) had ED. Multivariate logistic regression analysis revealed that eGDR was significantly and inversely associated with the risk of ED (odds ratios = 0.85, 95% confidence intervals [CI]: 0.78, 0.92). Subgroup analyses indicated that this association was more pronounced among individuals with higher educational attainment. RCS regression confirmed a linear inverse relationship. Furthermore, eGDR demonstrated superior discriminative performance for ED (area under the curve [AUC] = 0.705, 95% CI: 0.679, 0.731) compared with traditional IR and obesity indices. Mediation analysis showed that NLR mediated 10.3% (95% CI: 2.3%, 25.2%) of the total association between eGDR and ED.

**Conclusion:**

This study provides the first evidence of a significant inverse association between eGDR and ED prevalence, with NLR serving as a partial mediator.

## Popular scientific summary

- Lower estimated glucose disposal rate (a non-invasive insulin resistance indicator) is significantly associated with higher erectile dysfunction prevalence;
- Linear, more prominent in higher-educated men, outperforms traditional indices;
- Neutrophil-to-lymphocyte ratio-reflected inflammation mediates 10.3%.

Erectile dysfunction (ED) is defined as the persistent inability to achieve or maintain an erection sufficient for satisfactory sexual intercourse (1). It is a highly prevalent condition, particularly among men aged ≥ 40 years (2). Data from the Massachusetts Male Aging Study indicate that the overall prevalence of moderate, mild, and complete ED was 52% among men aged 40–70 years (3). ED may arise from organic or psychogenic factors, and both mechanisms frequently coexist in affected individuals (4). Numerous comorbidities and risk factors are significantly associated with ED, including physical inactivity, obesity, alcohol consumption, smoking, dyslipidemia, diabetes mellitus, hypogonadism, and hypertension. Furthermore, ED has been identified as a potential predictor of coronary heart disease (CHD), cardiovascular disease (CVD), and stroke (2, 5, 6). Collectively, ED imposes a substantial health burden on aging men. A substantial body of observational evidence has confirmed a significant association between ED and diabetes mellitus, with insulin resistance (IR) serving as a key underlying mechanism (7). Vascular endothelial dysfunction is pivotal in the pathogenesis of ED (8), and IR represents a primary driver of this process.

The estimated glucose disposal rate (eGDR), derived from readily obtainable clinical parameters – including waist circumference (WC), glycated hemoglobin A1c (HbA1c), and hypertension status – has been proposed as a straightforward surrogate marker of IR in individuals with type 1 diabetes mellitus (T1DM) (9). Previous research has extended the application of eGDR to patients with T2DM, acute ischemic stroke, and non-diabetic populations, demonstrating significant associations between eGDR and various outcomes, including diabetic complications, CVD, and all-cause mortality (10–13). However, the relationship between eGDR and ED remains inadequately characterized.

Given the established role of eGDR as an indicator of IR, we hypothesized that eGDR is inversely associated with the prevalence of ED. To investigate this relationship, we conducted a cross-sectional study using data from the National Health and Nutrition Examination Survey (NHANES).

## Methods

### Research population

The NHANES is a comprehensive, nationally representative research initiative overseen by the National Center for Health Statistics (NCHS) (14), designed to evaluate the interrelationships among disease prevention, health promotion, and nutrition. The survey employs a combination of structured interviews, physical examinations, and laboratory assessments to collect demographic, dietary, laboratory, and examination data.

**Fig. 1:**
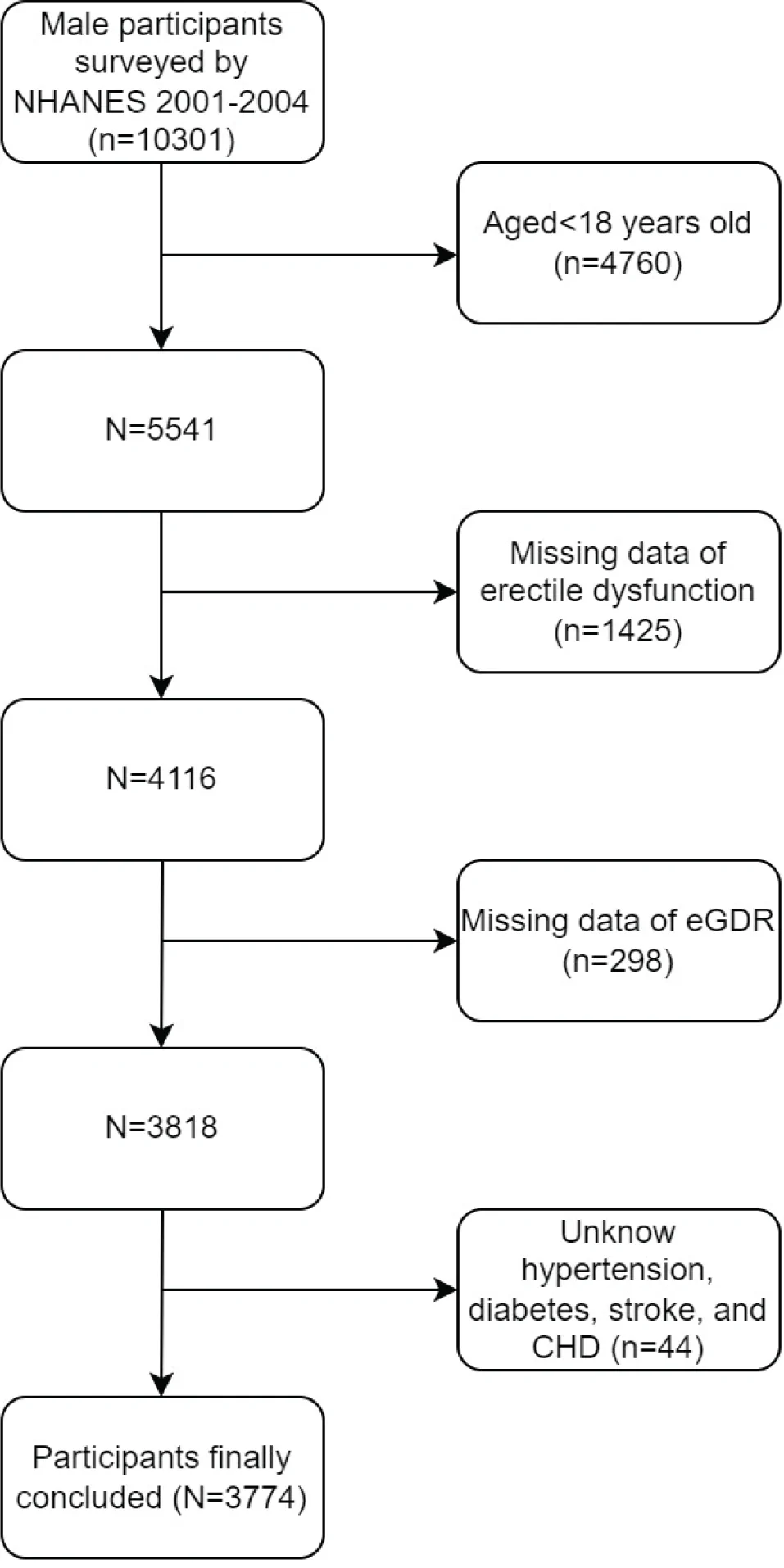
Flowchart of the sample selection from the 2001 to 2004 NHANES. eGDR: estimated glucose disposal rate; CHD: coronary heart disease.

This study utilized data from NHANES cycles conducted between 2001 and 2004. Male participants meeting the inclusion criteria were initially identified (*n* = 10,301). Exclusion criteria were applied sequentially: age < 18 years (*n* = 4,760), missing data on ED status (*n* = 1,425) or eGDR (*n* = 298), and missing information on hypertension, diabetes, stroke, or CHD (*n* = 44). The final analytical cohort comprised 3,774 subjects (Fig. 1).

### Calculation of IR and obesity surrogate markers

The following IR and obesity surrogate markers were calculated using simple anthropometric and biochemical measurements:

*eGDR (mg/kg/min) = 21.158 − (0.09 × WC [cm]) − (3.407 × hypertension) − (0.551 × HbA1c [%])*.

In this study, hypertension was coded as 0 (absent) or 1 (present) (15). Although the eGDR formula was originally developed for patients with T1DM, its application has been subsequently extended to patients with T2DM.

*METS-IR = BMI (kg/m^2^) × Ln[fasting TG (mg/dL) + (2 × fasting glucose [mg/dL])] / Ln[HDL-C (mg/dL)]* (16)

*TyG = Ln[fasting TG (mg/dL) × fasting glucose (mg/dL) / 2]* (17)

*Waist-to-height ratio (WHtR) = WC / height*

*Body mass index (BMI) = weight (kg) / height (m)^2^*

### Assessment of ED

ED was assessed using a single-item measure derived from the Massachusetts Male Aging Study (18). Participants were asked: ‘Many men encounter difficulties with sexual intercourse. How would you characterize your capacity to achieve and maintain an erection adequate for satisfactory intercourse?’ Response options included: ‘Usually able’, ‘Always or almost always able’, ‘Never able’, and ‘Sometimes able’. For analytical purposes, participants who responded ‘Never able’ or ‘Sometimes able’ were classified as having ED, whereas those who reported being ‘Usually able’ or ‘Always or almost always able’ were categorized as not having ED.

### Measurement of covariates

Based on previous studies (15, 16), the final analytical model incorporated potential confounding factors associated with both eGDR and ED. Demographic variables included age, race, height, weight, WC, educational attainment, physical activity, and poverty-to-income ratio (PIR). Questionnaire data encompassed smoking status, diabetes mellitus, alcohol consumption, stroke, CHD, and hypertension. Laboratory biomarkers included total cholesterol (TC), uric acid, low-density lipoprotein cholesterol (LDL-C), neutrophil count, triglycerides (TG), creatinine, lymphocyte count, high-density lipoprotein cholesterol (HDL-C), and albumin. The neutrophil-to-lymphocyte ratio (NLR) was calculated by dividing the absolute neutrophil count by the absolute lymphocyte count (19).

### Statistical analysis

Subjects were stratified into quartiles based on eGDR values (Q1: ≤ 6.00; Q2: 6.00–8.69; Q3: 8.69–9.88; Q4: ≥ 9.88). Mobile examination center (MEC) weights were used to obtain prevalence estimates that were representative of the US population. Given the data spanned two survey cycles from 2001 to 2004, MEC weights × 0.5 were applied as the final weights. The association between ED and eGDR was evaluated using weighted-multiple logistic regression models, with results presented as odds ratios (ORs) and 95% confidence intervals (CIs). Three distinct models were constructed: Model 1 (unadjusted); Model 2 (adjusted for race and age); and Model 3 (comprehensively adjusted for age, race, alcohol consumption, smoking, CHD, stroke, creatinine, PIR, educational level, TG, marital status, albumin, and uric acid). Multicollinearity was assessed using the variance inflation factor (VIF), with a VIF ≥ 5 indicating significant multicollinearity. None of the covariates exhibited multicollinearity (Table 1).

**Table 1:** Collinearity statistics

Variable	VIF
eGDR	1.16
Age	1.42
Race	1.56
PIR	1.44
Marital status	1.43
Educational level	1.78
CHD	1.09
Stroke	1.06
Smoking	1.09
Drinking	1.09
TG	1.06
Creatinine	1.11
Albumin	1.10
Uric acid	1.12

VIF: variance inflation factor; eGDR: estimated glucose disposal rate; PIR: poverty-to-income ratio; CHD: coronary heart disease; TG: triglycerides.

The potential influence of covariates on the eGDR–ED association was further explored through weighted subgroup and interaction analyses. The non-linear relationship between ED and eGDR was examined using weighted restricted cubic spline (RCS) curves. The proportion of the mediating effect attributable to NLR was quantified using weighted mediation analysis with the ‘mediation’ R package. Both the mediator model and the outcome model simultaneously controlled for the confounding factors included in Model 3. Using 1,000 non-parametric bootstrap resamples, the direct effect (DE), indirect effect (IE), and total effect (TE) were estimated, and the mediating proportion was calculated as (IE / TE × 100%). Given the observational design and cross-sectional assessment of variables, these assumptions cannot be formally tested and should be interpreted with caution.

Receiver operating characteristic (ROC) analysis was performed to evaluate the diagnostic performance of eGDR, HbA1c, METS-IR (metabolic score for insulin resistance), BMI (body mass index), TyG (triglyceride-glucose index), WHtR (waist to height ratio), and WC for ED. The DeLong test was used to compare area under the curves (AUCs) between markers. Incremental predictive value was assessed by comparing a baseline model (age only) with an eGDR-augmented model using: 1) C-statistic (DeLong test); 2) continuous net reclassification improvement (NRI); and 3) integrated discrimination improvement (IDI). In a sensitivity analysis, ED was redefined more stringently by including only participants who responded ‘Never able’ to maintain an erection. All statistical analyses were performed using Free Statistics software and R software.

## Results

### Baseline characteristics

A total of 3,774 subjects, ranging in age from 18 to 85 years, were included. Of these, 1,072 (28.4%) reported ED. Subjects with ED had significantly lower eGDR values than those without ED. Men with ED were more likely to be older, have lower educational attainment, be married or living with a partner, have a lower PIR, and exhibit higher BMI, WC, HbA1c, TG, and NLR. They also were more frequently former smokers, and had higher prevalences of diabetes, stroke, hypertension, and CHD (all *P* < 0.05). Comprehensive demographic and clinical characteristics are presented in Table 2.

**Table 2:** Characteristics of the study population based on erectile dysfunction, weighted

Characteristic	Overall *n* = 52,890,592 *N* = 3,774	No ED *n* = 42,425,981 *N* = 2,702	ED *n* = 10,464,611 *N* = 1,072	*P*
Age	45.45 (16.09)	41.51 (13.60)	61.40 (15.57)	< 0.001
Race, *n* (%)				0.188
Mexican American	4004342.7 (7.6)	3279360.2 (7.7)	724982.5 (6.9)	
Other Hispanic	2448573.6 (4.6)	1888061.7 (4.5)	560511.9 (5.4)	
Non-Hispanic White	39145467.8 (74.0)	31061742.1 (73.2)	8083725.7 (77.2)	
Non-Hispanic Black	5152273.1 (9.7)	4333347.4 (10.2)	818925.7 (7.8)	
Other race	2139935.5 (4.0)	1863470.1 (4.4)	276465.4 (2.6)	
Education level, *n* (%)				< 0.001
Less than high school	9282408.0 (17.6)	6121751.4 (14.4)	3160656.6 (30.2)	
High school or above	43564149.5 (82.4)	36260194.8 (85.6)	7303954.6 (69.8)	
Marital, *n* (%)				< 0.001
Married/living with partner	36950651.7 (69.9)	28806158.0 (67.9)	8144493.7 (77.8)	
Separated/divorced/widowed	6166316.3 (11.7)	4648846.0 (11.0)	1517470.2 (14.5)	
Never married	9769536.0 (18.5)	8966888.7 (21.1)	802647.3 (7.7)	
Alcohol status, *n* (%)				0.032
Current or ever, %	44751657.5 (84.6)	36187198.4 (85.3)	8564459.1 (81.8)	
Never	8119423.5 (15.4)	6219271.4 (14.7)	1900152.1 (18.2)	
Smoking status, *n* (%)				< 0.001
Current or ever, %	30252165.9 (57.2)	23201132.8 (54.7)	7051033.1 (67.4)	
Never	22632695.7 (42.8)	19221389.3 (45.3)	3411306.4 (32.6)	
Hypertension, *n* (%)				< 0.001
No	39024014.4 (73.8)	33678994.5 (79.4)	5345019.9 (51.1)	
Yes	13866578.3 (26.2)	8746986.9 (20.6)	5119591.3 (48.9)	
Diabetes, *n* (%)				< 0.001
No	48340647.6 (91.4)	40378144.8 (95.2)	7962502.8 (76.1)	
Yes	3953836.1 (7.5)	1600076.5 (3.8)	2353759.6 (22.5)	
CHD, *n* (%)				< 0.001
No	50169774.2 (94.9)	41296880.7 (97.3)	8872893.6 (84.8)	
Yes	2720818.4 (5.1)	1129100.8 (2.7)	1591717.6 (15.2)	
Stroke, *n* (%)				< 0.001
No	51862048.7 (98.1)	42152025.1 (99.4)	9710023.6 (92.8)	
Yes	1028543.9 (1.9)	273956.3 (0.6)	754587.6 (7.2)	
Body mass index, kg/m^2^	27.97 (5.33)	27.81 (5.26)	28.64 (5.58)	0.025
Waist circumference, cm	99.96 (14.59)	98.88 (14.33)	104.38 (14.78)	< 0.001
HbA1c, %	5.52 (0.90)	5.42 (0.75)	5.94 (1.24)	< 0.001
Albumin, g/dL	43.74 (2.91)	44.05 (2.83)	42.46 (2.90)	< 0.001
Total cholesterol, mmol/L	5.12 (4.45, 5.79)	5.15 (4.47, 5.84)	5.02 (4.40, 5.61)	0.053
Triglycerides, mmol/L	1.43 (0.99, 2.18)	1.39 (0.97, 2.09)	1.60 (1.12, 2.51)	< 0.001
HDL-cholesterol, mmol/L	1.16 (1.00, 1.40)	1.16 (0.98, 1.40)	1.19 (1.01, 1.41)	0.229
LDL-cholesterol, mmol/L	3.05 (2.46, 3.67)	3.08 (2.50, 3.70)	2.90 (2.31, 3.36)	< 0.001
Creatinine, umol/L	88.40 (79.56, 97.24)	88.40 (79.56, 97.24)	88.40 (79.56, 106.08)	0.024
Uric acid, umol/L	356.90 (309.30, 410.40)	356.90 (309.30, 410.40)	356.90 (309.30, 416.40)	0.535
PIR	3.25 (1.57)	3.32 (1.57)	2.98 (1.53)	< 0.001
NLR	2.25 (0.84)	2.18 (0.75)	2.51 (1.09)	< 0.001
eGDR	8.23 (2.39)	8.57 (2.20)	6.82 (2.62)	< 0.001

For continuous variables: survey-weighted mean (SD) or median (IQR). For categorical variables: survey-weighted *n* (%). HDL-c: High density lipoprotein cholesterol; LDL-c: Low density lipoprotein cholesterol; PIR: poverty-income ratio; eGDR: estimated glucose disposal rate; CHD: coronary heart disease; NLR: neutrophil-to-lymphocyte ratio; HbA1c: hemoglobin A1c.

### Association between eGDR and ED

**Fig. 2:**
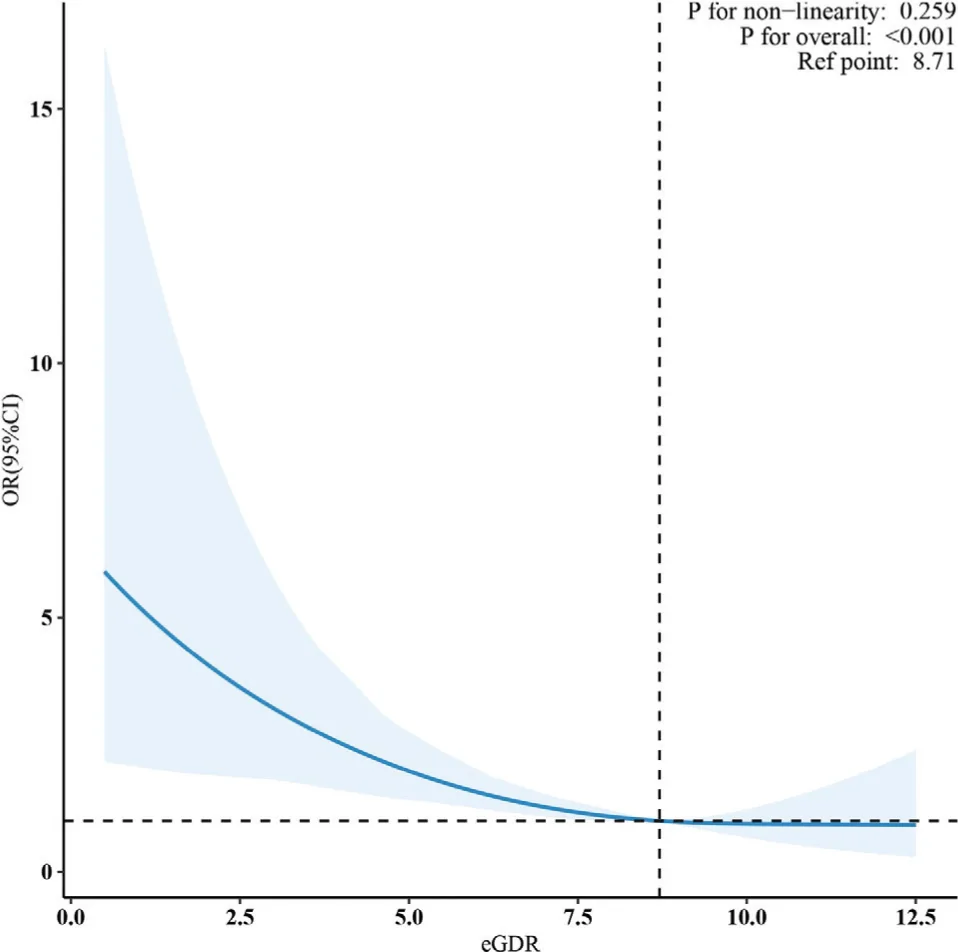
Restricted cubic spline fitting for the association between eGDR levels and ED. Adjusted for age, race, drinking, smoking, CHD, stroke, creatinine, PIR, education level, TG, marital status, albumin, and uric acid. eGDR: estimated glucose disposal rate; ED: erectile dysfunction; CI: confidence intervals; OR: odds ratios; PIR: poverty-income ratio; CHD: coronary heart disease; TG: triglycerides.

To investigate the association between eGDR and ED, three multivariate weighted logistic regression models were constructed (Table 3). Model 1 (unadjusted) identified a statistically significant inverse association between eGDR and ED. This inverse association persisted after comprehensive adjustment for all covariates in Model 3 (OR = 0.85, 95% CI: 0.78, 0.92). In the sensitivity analysis with eGDR categorized into quartiles, participants in the fourth quartiles demonstrated statistically significant 58% reductions in ED risk, compared with those in the lowest quartile (Model 3). RCS analysis illustrated a linear inverse association between eGDR and ED prevalence (Fig. 2).

**Table 3:** Weighted-multivariable logistic regression for the association between eGDR and ED

Subgroups	Model 1		Model 2		Model 3	
	OR (95% CI)	*P*	OR (95% CI)	*P*	OR (95% CI)	*P*
eGDR	0.74 (0.70, 0.78)	< 0.001	0.86 (0.81, 0.91)	< 0.001	0.85 (0.78, 0.92)	0.003
eGDR (category)						
Q1	1(Ref)		1(Ref)		1(Ref)	
Q2	0.47 (0.35, 0.64)	< 0.001	0.61 (0.43, 0.84)	< 0.001	0.72 (0.41, 1.28)	0.202
Q3	0.29 (0.20, 0.40)	< 0.001	0.38 (0.28, 0.51)	< 0.001	0.45 (0.25, 0.79)	0.015
Q4	0.12 (0.08, 0.18)	< 0.001	0.56 (0.35, 0.88)	0.016	0.42 (0.18, 0.96)	0.043
*P* for trend		< 0.001		< 0.001		0.001

Model 1: None covariates were adjusted; Model 2: race and age were adjusted; Model 3, age, race, drinking, smoking, CHD, stroke, creatinine, PIR, education level, TG, marital status, albumin, and uric acid were adjusted. eGDR: estimated glucose disposal rate; ED: erectile dysfunction; PIR: poverty-income ratio; CHD: coronary heart disease; TG: triglycerides; OR: odds ratios; CI: confidence intervals.

### Subgroup analysis

**Fig. 3:**
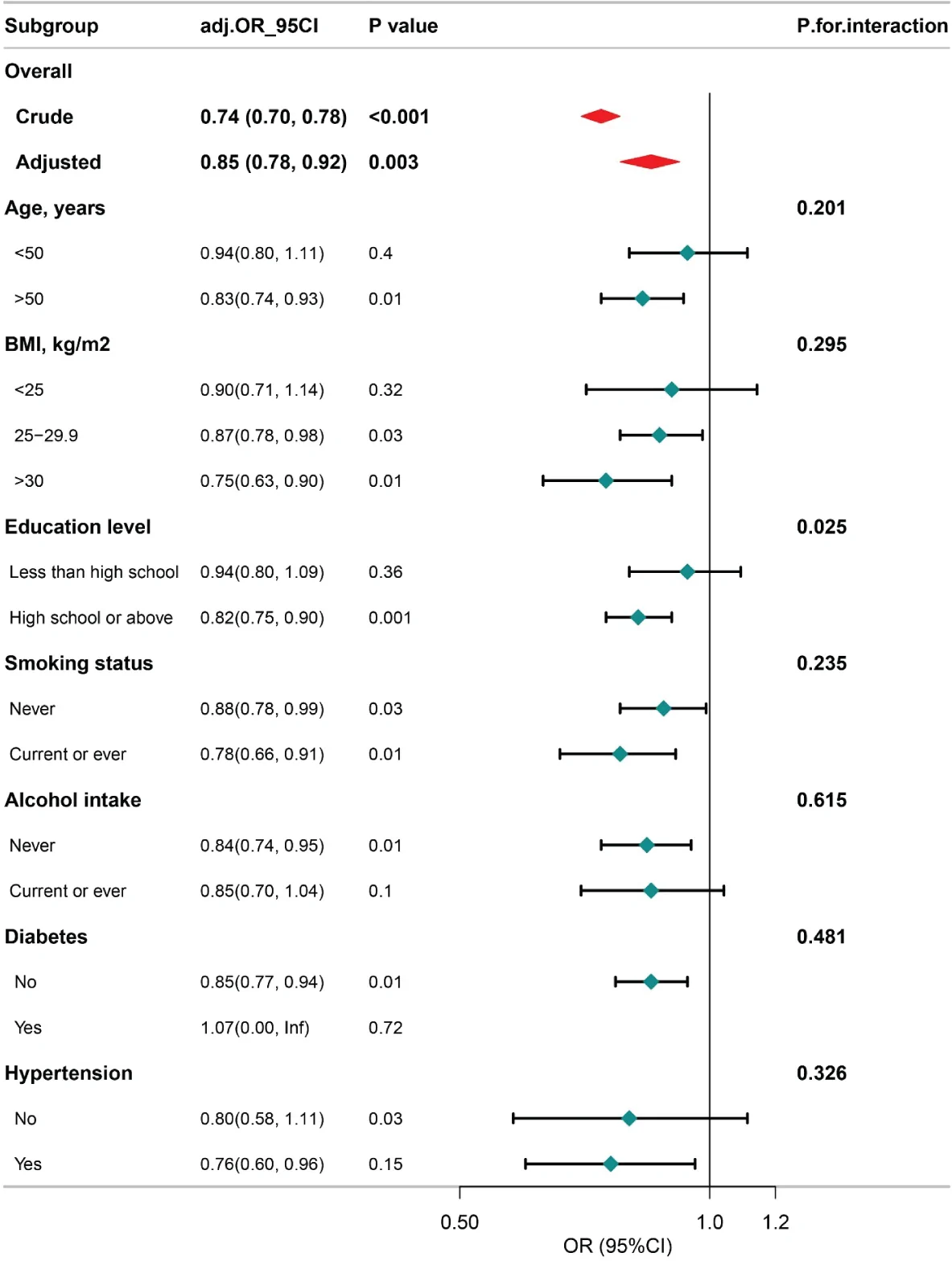
Association between eGDR and the risk of ED in various subgroups. eGDR: estimated glucose disposal rate; ED: erectile dysfunction; CI: confidence intervals; OR: odds ratios; BMI: body mass index.

The robustness of the eGDR–ED association was assessed through extensive interaction tests and subgroup analyses to identify potential effect modification across diverse subpopulations (Fig. 3). The results demonstrated a consistent significant association between eGDR and ED across most subgroups. Notably, this association was more pronounced among individuals with higher educational attainment.

### Mediating role of NLR

**Fig. 4:**
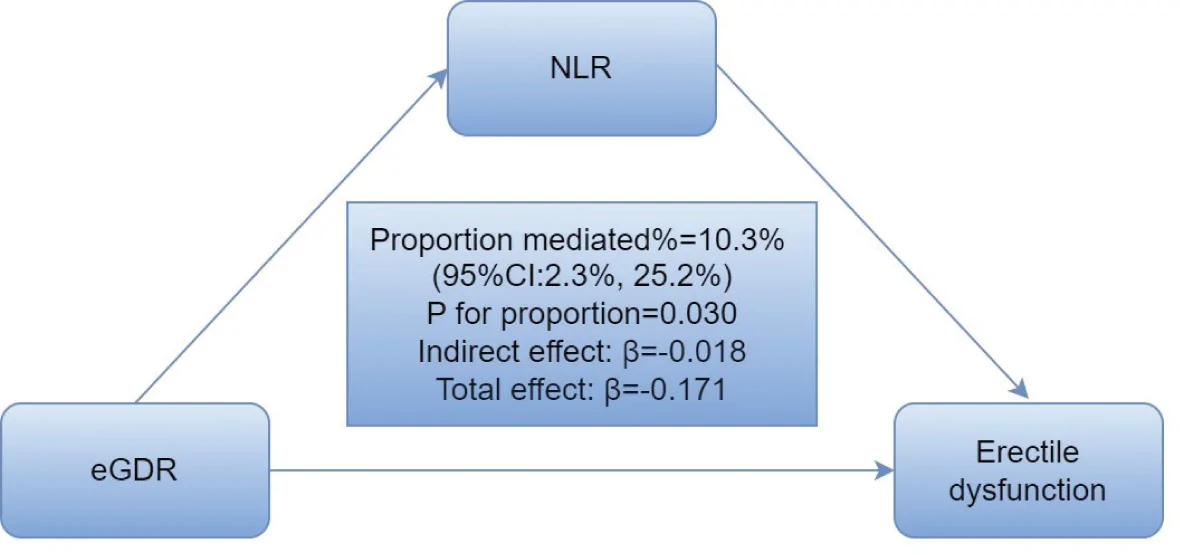
The mediating effect of NLR on the relationship between eGDR and ED. eGDR: estimated glucose disposal rate; ED: erectile dysfunction; CI: confidence intervals; NLR: neutrophil-to-lymphocyte ratio.

Mediation analysis confirmed the mediating role of NLR in the eGDR–ED association. The results indicated that 10.3% (95% CI: 2.3–25.2%) of the observed association between eGDR and ED risk was mediated through NLR (Fig. 4; Table 4).

**Table 4:** Mediation analysis of NLR in the association between eGDR and erectile dysfunction, weighted

Independent variable	Mediator	Total effect		Indirect effect		Direct effect		Proportion mediated, %
		Coefficient (95% CI)	*P*	Coefficient (95% CI)	*P*	Coefficient (95% CI)	*P*	
eGDR	NLR	-0.171 (-0.240, -0.100)	< 0.001	-0.018 (-0.036, -0.004)	0.030	-0.150 (-0.222, -0.079)	< 0.001	10.3

eGDR: estimated glucose disposal rate; NLR: neutrophil-to-lymphocyte ratio; CI: confidence intervals.

### Predictive value of eGDR for ED

**Fig. 5:**
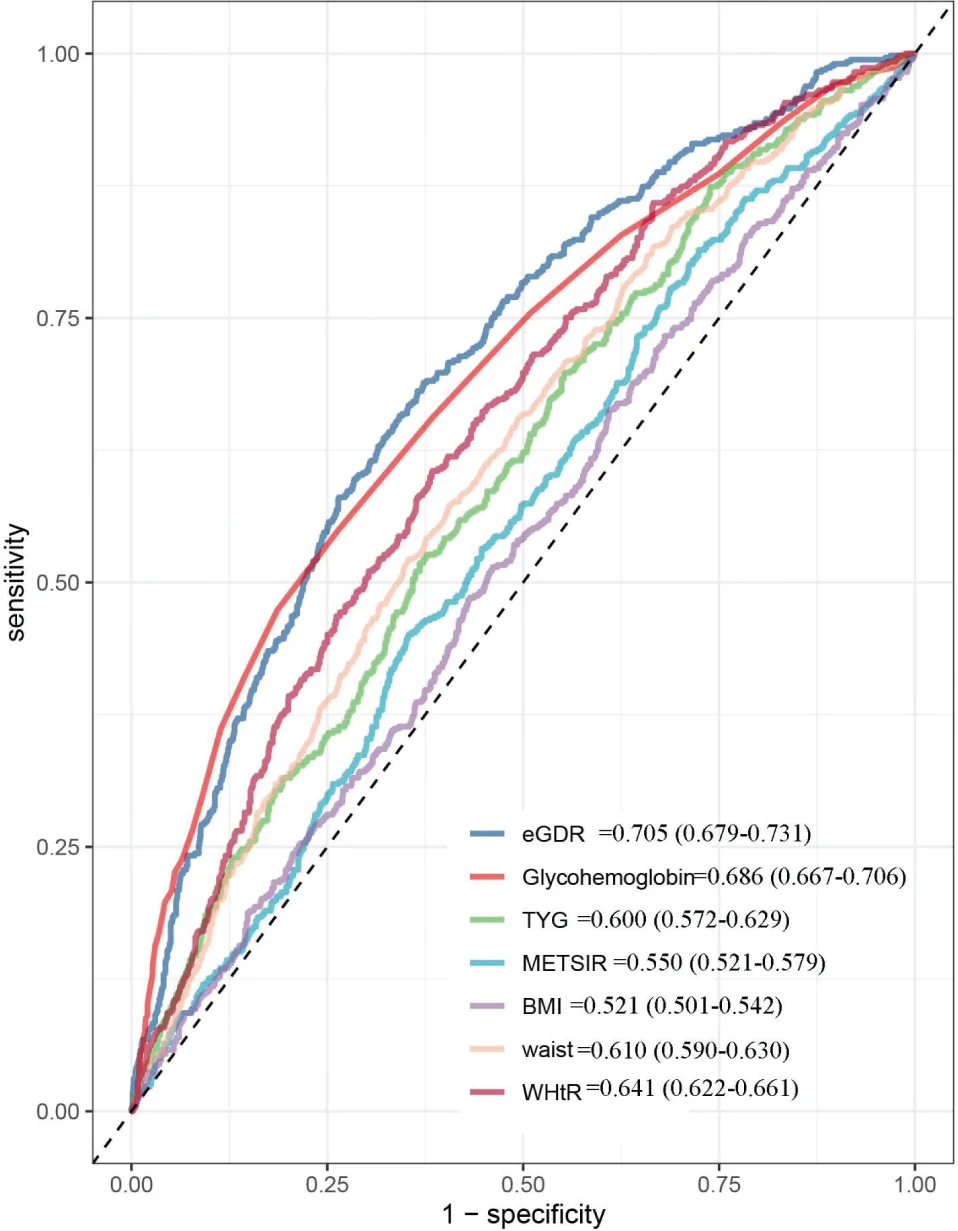
ROC analysis of eGDR, HbA1c, METS-IR, BMI, TyG, WHtR, and WC to ED among American adults. eGDR: estimated glucose disposal rate; HbA1c: hemoglobin A1c; METS-IR: metabolic score for insulin resistance; TyG: triglyceride-glucose index; BMI: body mass index; WC: waist circumference; WHtR: waist to height ratio; ED: erectile dysfunction; ROC: receiver operating characteristic.

The ROC curve analysis (Fig. 5) evaluated the diagnostic performance of eGDR, HbA1c, METS-IR, BMI, TyG, WHtR, and WC for ED. As shown in Table 5, eGDR exhibited the highest discrimination for ED, with an AUC of 0.705 (95% CI: 0.679–0.731), significantly outperforming other IR and obesity surrogate markers (*P* < 0.001).

**Table 5:** The AUC for each index to discriminate erectile dysfunction

Variable	AUC	95% CI	Cutoff value	Sensitivity	Specificity	Delong *P*-value
eGDR	0.705	0.679–0.731	8.48	0.689	0.627	Reference
HbA1c	0.686	0.667–0.706	5.55	0.559	0.728	< 0.001
METS-IR	0.550	0.521–0.579	45.26	0.451	0.646	< 0.001
TyG	0.600	0.572–0.629	8.92	0.518	0.632	< 0.001
BMI	0.521	0.501–0.542	31.78	0.226	0.821	< 0.001
WC	0.610	0.590–0.630	100.0	0.605	0.572	< 0.001
WHtR	0.641	0.622–0.661	0.576	0.601	0.606	< 0.001

eGDR: estimated glucose disposal rate; HbA1c: hemoglobin A1c; METS-IR: metabolic score for insulin resistance; TyG: triglyceride-glucose index; BMI: body mass index; WC: waist circumference; WHtR: waist to height ratio; CI: confidence intervals; AUC: area under the curve.

### Incremental predictive Value

**Fig. 6:**
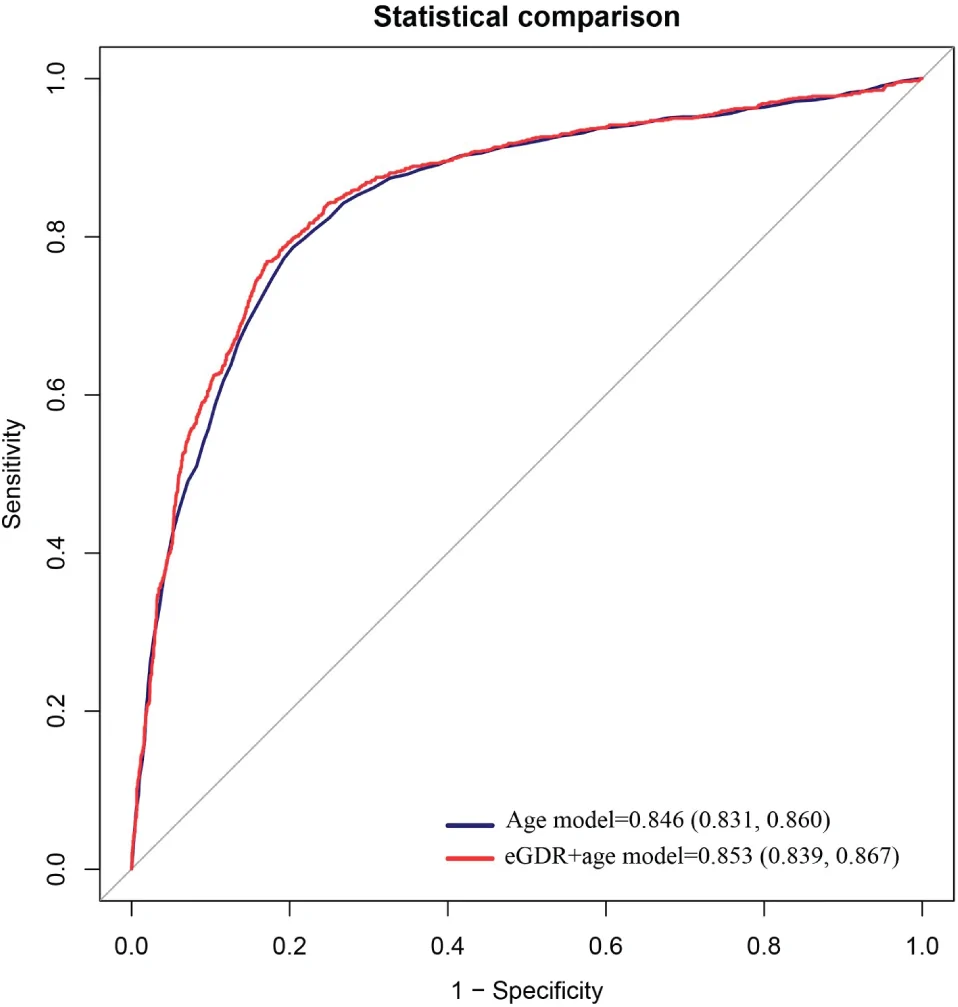
ROC curve analysis for age and eGDR combined age to identify ED. eGDR: estimated glucose disposal rate; ED: erectile dysfunction; ROC: receiver operating characteristic.

Incorporation of eGDR into the age-only model significantly improved discrimination: the C-statistic increased from 0.846 to 0.853 (DeLong test, *P* < 0.001). The categorical NRI for the combined eGDR–age model was 3.0% (*P* = 0.016), and the IDI was 1.7% (*P* < 0.001), demonstrating superior performance compared with the age-only model (Table 6 and Fig. 6).

**Table 6:** Incremental predictive value of eGDR for ED

Variable	*c*-statistic	*P*	NRI (95% CI)	*P*	IDI (95% CI)	*P*
Age	0.846 (0.831, 0.860)	1.0 (Ref)	1.0 (Ref)		1.0 (Ref)	
+eGDR	0.853 (0.839, 0.867)	< 0.001	0.030 (0.006, 0.055)	0.016	0.017 (0.011, 0.022)	< 0.001

NRI: net reclassification improvement; IDI: integrated discrimination improvement; eGDR: estimated glucose disposal rate; ED: erectile dysfunction; CI: confidence intervals.

### Sensitivity analysis

To minimize potential confounding and enhance the precision of our findings, we performed an additional sensitivity analysis with a more stringent definition of ED, including only participants who reported being ‘Never able’ to sustain an erection (Table 7). This analysis included 437 ED patients and 3,337 non-ED patients. The association between eGDR and ED remained statistically significant (OR = 0.86, 95% CI: 0.75–0.99).

**Table 7:** Weighted-multivariable logistic regression for the sensitivity analysis between eGDR and ED

Subgroups	Model 1		Model 2		Model 3	
	OR (95% CI)	*P*	OR (95% CI)	*P*	OR (95% CI)	*P*
eGDR	0.75 (0.70, 0.81)	< 0.001	0.64 (0.38, 1.05)	< 0.001	0.86 (0.75, 0.99)	0.041
eGDR (category)						
Q1	1(Ref)		1(Ref)		1(Ref)	
Q2	0.54 (0.35, 0.83)	0.007	0.64 (0.38, 1.05)	0.075	0.88 (0.43, 1.80)	0.660
Q3	0.38 (0.21, 0.68)	0.002	0.51 (0.14, 1.88)	0.243	0.71 (0.29, 1.73)	0.370
Q4	0.10 (0.05, 0.21)	< 0.001	0.58 (0.28, 1.21)	0.141	0.50 (0.31, 0.78)	0.004
*P* for trend		< 0.001		0.044		0.006

Model 1: None covariates were adjusted; Model 2: race and age were adjusted; Model 3, age, race, drinking, smoking, CHD, stroke, creatinine, PIR, education level, TG, marital status, albumin, and uric acid were adjusted. eGDR: estimated glucose disposal rate; ED: erectile dysfunction; CI: confidence intervals; OR: odds ratios; PIR: poverty-income ratio; CHD: coronary heart disease; TG: triglycerides.

## Discussion

In this cross-sectional study of a nationally representative sample of 3,774 participants, we identified a significant inverse association between eGDR and ED, which was particularly pronounced among individuals with higher educational attainment. Mediation analysis further indicated that NLR partially mediated the eGDR–ED association. Moreover, among the assessed surrogate indices for IR and obesity – including eGDR, HbA1c, METS-IR, TyG, BMI, WC, and WHtR – eGDR demonstrated the highest AUC for predicting ED risk.

Numerous studies have demonstrated an association between IR and the development of ED. A cross-sectional study reported that patients with IR are at significantly increased risk of ED, even after adjustment for confounding variables (20). The hyperinsulinemic–euglycemic clamp (HIEC) is widely regarded as the gold standard for assessing IR (21). However, this method requires frequent arterial blood glucose measurements, which limits its practical applicability (22, 23). Consequently, there is a need for more straightforward and feasible approaches to assess IR in clinical and epidemiological settings.

The eGDR, incorporating readily accessible clinical parameters such as WC, HbA1c, and hypertension status, has been proposed as a simple surrogate marker of IR in individuals with T1DM (9). Prior research has indicated that this alternative method exhibits a high degree of accuracy when compared with gold standard measures (9, 24). eGDR has been identified as an independent predictor of coronary artery disease (25), depression (15), all-cause mortality (26), and peripheral vascular disease (27) in patients with T1DM (28–31). Recent studies have extended the investigation of eGDR’s applicability to non-diabetic patients (32), patients with T2DM (24, 33), and those with acute ischemic stroke (24, 34). Given the shared risk factors between ED and CVD (35), it is plausible to hypothesize an association between eGDR and ED. To our knowledge, this study is the first to investigate this association. Furthermore, the significantly greater AUC for eGDR compared with other IR and obesity surrogate markers indicates its potential clinical utility.

Subgroup analysis revealed that the eGDR–ED association was significantly more pronounced in individuals with higher educational attainment (*P* for interaction < 0.001). Previous research has indicated that individuals with sedentary lifestyles and lower educational attainment are more prone to ED (36, 37). Lower educational level is recognized as a risk factor for ED and may exert a greater influence on ED risk than IR within this population. Consequently, the impact of IR on ED prevalence may be relatively attenuated in individuals with lower educational levels. Conversely, in more educated populations, who are generally considered healthier and have fewer other potential risk factors, the association between IR and ED is anticipated to be more pronounced. However, educational level is highly correlated with age, which is a well-established independent risk factor for both ED and IR. Therefore, it remains uncertain whether the observed discrepancy stems from the independent effect of education per se, the confounding influence of age, or unmeasured health-related behaviors.

Our findings indicate that NLR partially mediates the association between IR, as measured by eGDR, and ED, highlighting the importance of monitoring NLR levels in patients with low eGDR. Previous studies have established a significant association between ED and chronic inflammation (38, 39). Consequently, modulating NLR – particularly through reducing neutrophil count and increasing lymphocyte count – may decrease the risk of ED in individuals with low eGDR. These findings enhance our understanding of the complex interrelationships among IR, inflammation, and ED. The cross-sectional nature of biomarker assessment, in which exposure and mediating factors were measured concurrently, and the observational design preclude the establishment of temporal or causal sequences. Therefore, the above interpretation should be regarded as a biologically plausible hypothesis rather than a definitive mechanistic inference.

Two recent studies have utilized the same NHANES 2001–2004 dataset to investigate correlates of ED. Liu et al. reported that NLR was positively associated with ED (OR = 1.35, 95% CI: 1.09–1.68) among 3,610 participants, with monocyte-to-lymphocyte ratio (MLR) demonstrating the highest discriminative accuracy (AUC = 0.616) among inflammatory markers (39). Li et al. identified a positive association between the TyG index and ED prevalence in males aged 20–70 years (7). Our study extends these findings in several important respects. Firstly, whereas Liu et al. treated NLR as the primary exposure, our study positions NLR as a mediator in the eGDR–ED pathway, quantifying its mediating proportion at 10.3%. This reframes NLR not merely as an independent predictor but as a partial biological intermediary linking IR to ED. Secondly, the eGDR exhibited markedly superior discriminative performance (AUC = 0.705) compared with both the inflammatory markers evaluated by Liu et al. (AUC = 0.616 for MLR) and the TyG index examined by Li et al., suggesting that eGDR may be a more clinically useful screening tool. These distinctions underscore that while chronic inflammation (as reflected by NLR) and lipid–glucose metabolism (as reflected by TyG) are both relevant to ED pathophysiology, eGDR – by integrating WC, glycemic control, and hypertension status – captures a more comprehensive metabolic profile with greater predictive value.

Several mechanisms may underlie the association between ED and IR. IR is known to enhance the production of inflammatory cytokines and oxidative stress in endothelial cells (40), resulting in significant depletion of NO in tissues exposed to free radicals. The reduction in NO availability subsequently impairs endothelial function (41, 42). Furthermore, endothelin-B receptors have been implicated in elevated reactive oxygen species levels, endothelial dysfunction, and increased vasoconstriction within erectile tissues. Notably, IR has been shown to upregulate endothelin-B receptor expression in the vasculature of obese rats with IR (43). This alteration in endothelial function exacerbates the decline in insulin metabolism, creating a negative feedback loop (21). IR is indicative of pre-diabetes, and its progression toward diabetes parallels the progression of endothelial dysfunction into atherosclerosis (44). Moreover, IR is associated with elevated basal serum insulin levels, which subsequently activate the sympathetic nervous system and increase atherosclerotic risk factors, collectively contributing to ED (45, 46). IR also elevates endothelin-1 levels – a potent vasoconstrictor affecting both arterial and venous systems – within penile cavernous tissues (47). Under conditions of IR, Leydig cells in the testes produce reduced amounts of testosterone, thereby contributing to ED (48).

This study has several notable strengths. It provides the first investigation of the association between eGDR and ED, while also evaluating its predictive performance for ED relative to various obesity and IR indicators. In addition, we meticulously adjusted for potential confounding variables in the multivariate logistic regression analyses. However, several limitations should be acknowledged. The cross-sectional design inherently precludes causal inference regarding the eGDR–ED association. Moreover, ED was assessed using a self-report questionnaire; although previous studies have confirmed its validity, the severity of ED was not quantified. Erectile function was not assessed using the International Index of Erectile Function-5, which may have introduced sample selection bias. Furthermore, because this study was conducted exclusively in a US sample, additional investigations are needed to determine the generalizability of these findings to populations in diverse geographical regions. Finally, the relatively low prevalence of ED may have resulted in diminished statistical power for certain subgroup analyses.

## Conclusion

This study revealed a significant inverse association between eGDR and ED. Moreover, eGDR demonstrated superior discrimination for ED compared with other surrogate markers of obesity and IR.

## Declarations

### Availability of data and materials

All raw data were publicly available at the NHANES database (https://www.cdc.gov/nchs/nhanes/index.htm).

## References

[1] Yafi FA, Jenkins L, Albersen M, Corona G, Isidori AM, Goldfarb S, et al. Erectile dysfunction. Nat Rev Dis Primers 2016; 2: 16003. doi: 10.1038/nrdp.2016.3 27188339 PMC5027992

[2] Shamloul R, Ghanem H. Erectile dysfunction. Lancet 2013; 381(9861): 153–65. doi: 10.1016/S0140-6736(12)60520-0 23040455

[3] Feldman HA, Goldstein I, Hatzichristou DG, Krane RJ, McKinlay JB. Impotence and its medical and psychosocial correlates: results of the Massachusetts Male Aging Study. J Urol 1994; 151(1): 54–61. doi: 10.1016/S0022-5347(17)34871-1 8254833

[4] Muneer A, Kalsi J, Nazareth I, Arya M. Erectile dysfunction. BMJ 2014; 348: g129. doi: 10.1136/bmj.g129 24468580

[5] Rosen RC, Wing R, Schneider S, Gendrano N, 3rd. Epidemiology of erectile dysfunction: the role of medical comorbidities and lifestyle factors. Urol Clin North Am 2005; 32(4): 403–17, v. doi: 10.1016/j.ucl.2005.08.004 16291033

[6] Dong JY, Zhang YH, Qin LQ. Erectile dysfunction and risk of cardiovascular disease: meta-analysis of prospective cohort studies. J Am Coll Cardiol 2011; 58(13): 1378–85. doi: 10.1016/j.jacc.2011.06.024 21920268

[7] Li L, Yao H, Dai W, Chen Y, Liu H, Ding W, et al. A higher TyG index is related with a higher prevalence of erectile dysfunction in males between the ages 20–70 in the United States, according to a cross-sectional research. Front Endocrinol (Lausanne) 2022; 13: 988257. doi: 10.3389/fendo.2022.988257 36157467 PMC9497651

[8] Billups KL, Bank AJ, Padma-Nathan H, Katz S, Williams R. Erectile dysfunction is a marker for cardiovascular disease: results of the minority health institute expert advisory panel. J Sex Med 2005; 2(1): 40–50; discussion 50–2. doi: 10.1111/j.1743-6109.2005.20104_1.x 16422903

[9] Epstein EJ, Osman JL, Cohen HW, Rajpathak SN, Lewis O, Crandall JP. Use of the estimated glucose disposal rate as a measure of insulin resistance in an urban multiethnic population with type 1 diabetes. Diabetes Care 2013; 36(8): 2280–5. doi: 10.2337/dc12-1693 23596179 PMC3714518

[10] Meng C, Xing Y, Huo L, Ma H. Relationship between estimated glucose disposal rate and type 2 diabetic retinopathy. Diabetes Metab Syndr Obes 2023; 16: 807–18. doi: 10.2147/DMSO.S395818 36959899 PMC10028301

[11] Ren X, Jiang M, Han L, Zheng X. Estimated glucose disposal rate and risk of cardiovascular disease: evidence from the China Health and Retirement Longitudinal Study. BMC Geriatr 2022; 22(1): 968. doi: 10.1186/s12877-022-03689-x 36517754 PMC9753298

[12] Kong X, Wang W. Estimated glucose disposal rate and risk of cardiovascular disease and mortality in U.S. adults with prediabetes: a nationwide cross-sectional and prospective cohort study. Acta Diabetol 2024; 61(11): 1413–21. doi: 10.1007/s00592-024-02305-1 38805079

[13] Lu Z, Xiong Y, Feng X, Yang K, Gu H, Zhao X, et al. Insulin resistance estimated by estimated glucose disposal rate predicts outcomes in acute ischemic stroke patients. Cardiovasc Diabetol 2023; 22(1): 225. doi: 10.1186/s12933-023-01925-1 37633905 PMC10464388

[14] Zipf G, Chiappa M, Porter KS, Ostchega Y, Lewis BG, Dostal J. National health and nutrition examination survey: plan and operations, 1999–2010. Vital Health Stat 1 2013; (56): 1–37. 25078429

[15] Chen Y, Lin H, Xu J, Zhou X. Estimated glucose disposal rate is correlated with increased depression: a population-based study. BMC Psychiatry 2024; 24(1): 786. doi: 10.1186/s12888-024-06257-2 39529068 PMC11556201

[16] Li C, Xu J. Negative correlation between metabolic score for insulin resistance index and testosterone in male adults. Diabetol Metab Syndr 2024; 16(1): 113. doi: 10.1186/s13098-024-01353-5 38783379 PMC11112955

[17] Huang J, Rozi R, Ma J, Fu B, Lu Z, Liu J, et al. Association between higher triglyceride glucose index and increased risk of osteoarthritis: data from NHANES 2015–2020. BMC Public Health 2024; 24(1): 758. doi: 10.1186/s12889-024-18272-9 38468219 PMC10929152

[18] Derby CA, Araujo AB, Johannes CB, Feldman HA, McKinlay JB. Measurement of erectile dysfunction in population-based studies: the use of a single question self-assessment in the Massachusetts Male Aging Study. Int J Impot Res 2000; 12(4): 197–204. doi: 10.1038/sj.ijir.3900542 11079360

[19] Zhang X, Wei R, Wang X, Zhang W, Li M, Ni T, et al. The neutrophil-to-lymphocyte ratio is associated with all-cause and cardiovascular mortality among individuals with hypertension. Cardiovasc Diabetol 2024; 23(1): 117. doi: 10.1186/s12933-024-02191-5 38566082 PMC10985955

[20] Russo GI, Cimino S, Fragala E, Privitera S, La Vignera S, Condorelli R, et al. Insulin resistance is an independent predictor of severe lower urinary tract symptoms and of erectile dysfunction: results from a cross-sectional study. J Sex Med 2014; 11(8): 2074–82. doi: 10.1111/jsm.12587 24836928

[21] Vasques AC, Novaes FS, de Oliveira Mda S, Souza JR, Yamanaka A, Pareja JC, et al. TyG index performs better than HOMA in a Brazilian population: a hyperglycemic clamp validated study. Diabetes Res Clin Pract 2011; 93(3): e98–100. doi: 10.1016/j.diabres.2011.05.030 21665314

[22] Bergman RN, Finegood DT, Ader M. Assessment of insulin sensitivity in vivo. Endocr Rev 1985; 6(1): 45–86. doi: 10.1210/edrv-6-1-45 3884329

[23] DeFronzo RA, Tobin JD, Andres R. Glucose clamp technique: a method for quantifying insulin secretion and resistance. Am J Physiol 1979; 237(3): E214–23. doi: 10.1152/ajpendo.1979.237.3.E214 382871

[24] Zabala A, Darsalia V, Lind M, Svensson AM, Franzen S, Eliasson B, et al. Estimated glucose disposal rate and risk of stroke and mortality in type 2 diabetes: a nationwide cohort study. Cardiovasc Diabetol 2021; 20(1): 202. doi: 10.1186/s12933-021-01394-4 34615525 PMC8495918

[25] Pane A, Conget I, Boswell L, Ruiz S, Vinals C, Perea V, et al. Insulin resistance is associated with preclinical carotid atherosclerosis in patients with type 1 diabetes. Diabetes Metab Res Rev 2020; 36(7): e3323. doi: 10.1002/dmrr.3323 32266782

[26] Nystrom T, Holzmann MJ, Eliasson B, Svensson AM, Sartipy U. Estimated glucose disposal rate predicts mortality in adults with type 1 diabetes. Diabetes Obes Metab 2018; 20(3): 556–63. doi: 10.1111/dom.13110 28884949

[27] Olson JC, Erbey JR, Forrest KY, Williams K, Becker DJ, Orchard TJ. Glycemia (or, in women, estimated glucose disposal rate) predict lower extremity arterial disease events in type 1 diabetes. Metabolism 2002; 51(2): 248–54. doi: 10.1053/meta.2002.30021 11833057

[28] Dastjerdi P, Hosseini Mohammadi NS, Anaraki N, Rahmati S, Nikfar R, Momeni S, et al. Estimated glucose disposal rate and risk of cardiovascular events in type 1 diabetes: a systematic review and meta-analysis. Diabetol Metab Syndr 2025; 17(1): 348. doi: 10.1186/s13098-025-01900-8 40841970 PMC12369242

[29] Vergani M, Borella ND, Rizzo M, Conti M, Perra S, Bianconi E, et al. Metabolic dysfunction-associated steatotic liver disease, insulin sensitivity and continuous glucose monitoring metrics in patients with type 1 diabetes: a multi-centre cross-sectional study. Diabetes Obes Metab 2025; 27(6): 3201–11. doi: 10.1111/dom.16333 40083078 PMC12046442

[30] Canat MM, Altuntas Y. Comparison of two estimated glucose disposal rate methods for detecting insulin resistance in adults with type 1 diabetes mellitus. Metab Syndr Relat Disord 2024; 22(4): 295–301. doi: 10.1089/met.2023.0217 38546845

[31] Nishtala R, Kietsiriroje N, Karam M, Ajjan RA, Pearson S. Estimated glucose disposal rate demographics and clinical characteristics of young adults with type 1 diabetes mellitus: a cross-sectional pilot study. Diab Vasc Dis Res 2020; 17(5): 1479164120952321. doi: 10.1177/1479164120952321 32883101 PMC7919212

[32] Liu C, Liu X, Ma X, Cheng Y, Sun Y, Zhang D, et al. Predictive worth of estimated glucose disposal rate: evaluation in patients with non-ST-segment elevation acute coronary syndrome and non-diabetic patients after percutaneous coronary intervention. Diabetol Metab Syndr 2022; 14(1): 145. doi: 10.1186/s13098-022-00915-9 36203208 PMC9535978

[33] Penno G, Solini A, Orsi E, Bonora E, Fondelli C, Trevisan R, et al. Insulin resistance, diabetic kidney disease, and all-cause mortality in individuals with type 2 diabetes: a prospective cohort study. BMC Med 2021; 19(1): 66. doi: 10.1186/s12916-021-01936-3 33715620 PMC7962330

[34] Xuan J, Juan D, Yuyu N, Anjing J. Impact of estimated glucose disposal rate for identifying prevalent ischemic heart disease: findings from a cross-sectional study. BMC Cardiovasc Disord 2022; 22(1): 378. doi: 10.1186/s12872-022-02817-0 35987992 PMC9392437

[35] Nehra A, Jackson G, Miner M, Billups KL, Burnett AL, Buvat J, et al. The Princeton III consensus recommendations for the management of erectile dysfunction and cardiovascular disease. Mayo Clin Proc 2012; 87(8): 766–78. doi: 10.1016/j.mayocp.2012.06.015 22862865 PMC3498391

[36] Yannas D, Zago E, Cavallini E, Todisco T, Vignozzi L, Corona G, et al. Education degree predicts cardiovascular outcomes in men suffering from erectile dysfunction. Andrology 2023; 11(6): 1086–95. doi: 10.1111/andr.13389 36642862

[37] Heidelbaugh JJ. Management of erectile dysfunction. Am Fam Physician 2010; 81(3): 305–12. 20112889

[38] Feng X, Mei Y, Wang X, Cui L, Xu R. Association between neutrophil to lymphocyte ratio and erectile dysfunction among US males: a population-based cross-sectional study. Front Endocrinol (Lausanne) 2023; 14: 1192113. doi: 10.3389/fendo.2023.1192113 37424870 PMC10326541

[39] Liu H, Dong H, Guo M, Cheng H. Association between inflammation indicators (MLR, NLR, SII, SIRI, and AISI) and erectile dysfunction in US adults: NHANES 2001–2004. J Health Popul Nutr 2024; 43(1): 169. doi: 10.1186/s41043-024-00667-4 39462407 PMC11514745

[40] Moon KH, Park SY, Kim YW. Obesity and erectile dysfunction: from bench to clinical implication. World J Mens Health 2019; 37(2): 138–47. doi: 10.5534/wjmh.180026 30079640 PMC6479091

[41] Aljada A, Dandona P. Effect of insulin on human aortic endothelial nitric oxide synthase. Metabolism 2000; 49(2): 147–50. doi: 10.1016/S0026-0495(00)91039-4 10690935

[42] Cersosimo E, DeFronzo RA. Insulin resistance and endothelial dysfunction: the road map to cardiovascular diseases. Diabetes Metab Res Rev 2006; 22(6): 423–36. doi: 10.1002/dmrr.634 16506274

[43] Katakam PV, Pollock JS, Pollock DM, Ujhelyi MR, Miller AW. Enhanced endothelin-1 response and receptor expression in small mesenteric arteries of insulin-resistant rats. Am J Physiol Heart Circ Physiol 2001; 280(2): H522–7. doi: 10.1152/ajpheart.2001.280.2.H522 11158947

[44] Chen S, Wu R, Huang Y, Zheng F, Ou Y, Tu X, et al. Insulin resistance is an independent determinate of ED in young adult men. PLoS One 2013; 8(12): e83951. doi: 10.1371/journal.pone.0083951 24391852 PMC3877124

[45] Musicki B, Hannan JL, Lagoda G, Bivalacqua TJ, Burnett AL. Mechanistic link between erectile dysfunction and systemic endothelial dysfunction in type 2 diabetic rats. Andrology 2016; 4(5): 977–83. doi: 10.1111/andr.12218 27153512 PMC4988920

[46] Kara E, Kahraman E, Dayar E, Yetik Anacak G, Demir O, Gidener S, et al. The role of resistin on metabolic syndrome-induced erectile dysfunction and the possible therapeutic effect of Boldine. Andrology 2020; 8(6): 1728–35. doi: 10.1111/andr.12853 32609430

[47] Francavilla S, Properzi G, Bellini C, Marino G, Ferri C, Santucci A. Endothelin-1 in diabetic and nondiabetic men with erectile dysfunction. J Urol 1997; 158(5): 1770–4. doi: 10.1016/S0022-5347(01)64125-9 9334598

[48] Pitteloud N, Hardin M, Dwyer AA, Valassi E, Yialamas M, Elahi D, et al. Increasing insulin resistance is associated with a decrease in Leydig cell testosterone secretion in men. J Clin Endocrinol Metab 2005; 90(5): 2636–41. doi: 10.1210/jc.2004-2190 15713702

